# Analysis of transcriptional modules during human fibroblast ageing

**DOI:** 10.1038/s41598-020-76117-y

**Published:** 2020-11-05

**Authors:** Yaelim Lee, G. V. Shivashankar

**Affiliations:** 1 Mechanobiology Institute, National University of Singapore, Singapore, Singapore; 2 FIRC Institute for Molecular Oncology (IFOM), Milan, Italy; 3 Department of Health Sciences and Technology (D-HEST), ETH Zurich, Zürich, Switzerland; 4 Paul Scherrer Institute, Villigen, Switzerland

**Keywords:** Cell biology, Computational biology and bioinformatics, Biomarkers

## Abstract

For systematic identification of transcription signatures of human cell aging, we carried out Weighted Gene Co-expression Network Analysis (WGCNA) with the RNA-sequencing data generated with young to old human dermal fibroblasts. By relating the modules to the donor's traits, we uncovered the natural aging- and premature aging disease-associated modules. The STRING functional association networks built with the core module memberships provided a systematic overview of genome-wide transcriptional changes upon aging. We validated the selected candidates via quantitative reverse transcription PCR (RT-qPCR) assay with young and aged human fibroblasts, and uncovered several genes involved in ECM, cell, and nuclear mechanics as a potential aging biomarker. Collectively, our study not only provides a snapshot of functional changes during human fibroblast aging but also presents potential aging markers that are relevant to cell mechanics.

## Introduction

Aging is a progressive loss of fitness, driven by damage accumulation, in living organisms across their life span. Assessing aging and anti-aging processes in cells or tissues is mainly performed by quantifying key biochemical molecules whose functions are characterized in 9 aging hallmarks-contexts: genomic instability, telomere attrition, epigenetic alterations, loss of proteostasis, deregulated nutrient sensing, mitochondrial dysfunction, cellular senescence, stem cell exhaustion, and altered intercellular communication^1^.

Importantly, aging is also accompanied by various biophysical changes in extracellular matrix (ECM), cell, and nucleus^2,3^. Our recent study demonstrated that the fibroblasts via nuclear reprogramming and re-differentiation by mechanical constraints showed higher contractility and enhanced ECM remodeling, compared to the control fibroblasts, and these characteristics were reminiscent of cellular rejuvenation^4^. This underscores the importance of changes in the mechanical state of the cells with aging. Nonetheless, little effort has been made for identifying aging biomarkers that can represent biophysical changes in the cells.

A comprehensive set of transcriptome data from various age groups was generated in a recent study^5^. This dataset is unique because, firstly, it covers all ages ranging from 1- to 96-year, which addresses a major weakness of previous studies where limited age groups were included. Secondly, this dataset includes 133 healthy individuals and 10 Hutchinson–Gilford progeria syndrome (HGPS) patients, enabling us to study natural aging as well as a premature aging disease caused by mutations of the *LMNA* gene, encoding lamin-A/C that plays a crucial role in nuclear mechanics^6^. Lastly, the entire transcriptomes were generated with one cell type, human dermal fibroblasts, which is an ideal model system for studying aging because they possess fundamental elements of aging such as cellular heterogeneity, stem cell population, and interactions with ECM^7,8^.

Hence, in this study, we took advantage of this dataset to identify candidate aging biomarkers for human cell aging and carried out Weighted Gene Co-expression Network Analysis (WGCNA), a network-based gene screening method^9^. We identified 8 modules, each consisting of highly interconnected genes in the co-expression network. By relating the modules to the donor's traits, namely age and HGPS, we uncovered the existence of aging-associated modules: 4 age-dependent modules and 1 HGPS-associated module. In these modules, we prioritized the core module memberships (i.e., genes), and used them to characterize the biological function of the modules by STRING, a database of known and predicted protein–protein interactions^10^. Further, we selected top candidate genes by considering the degree of changes in expression with age. Interestingly, several genes involved in ECM, cell, and nuclear mechanics were identified, and subsequently validated via RT-qPCR assay with young and aged human dermal fibroblasts.

## Results

### Weighted gene co-expression network analysis (WGCNA) of young to old dermal fibroblasts

**Figure 1:**
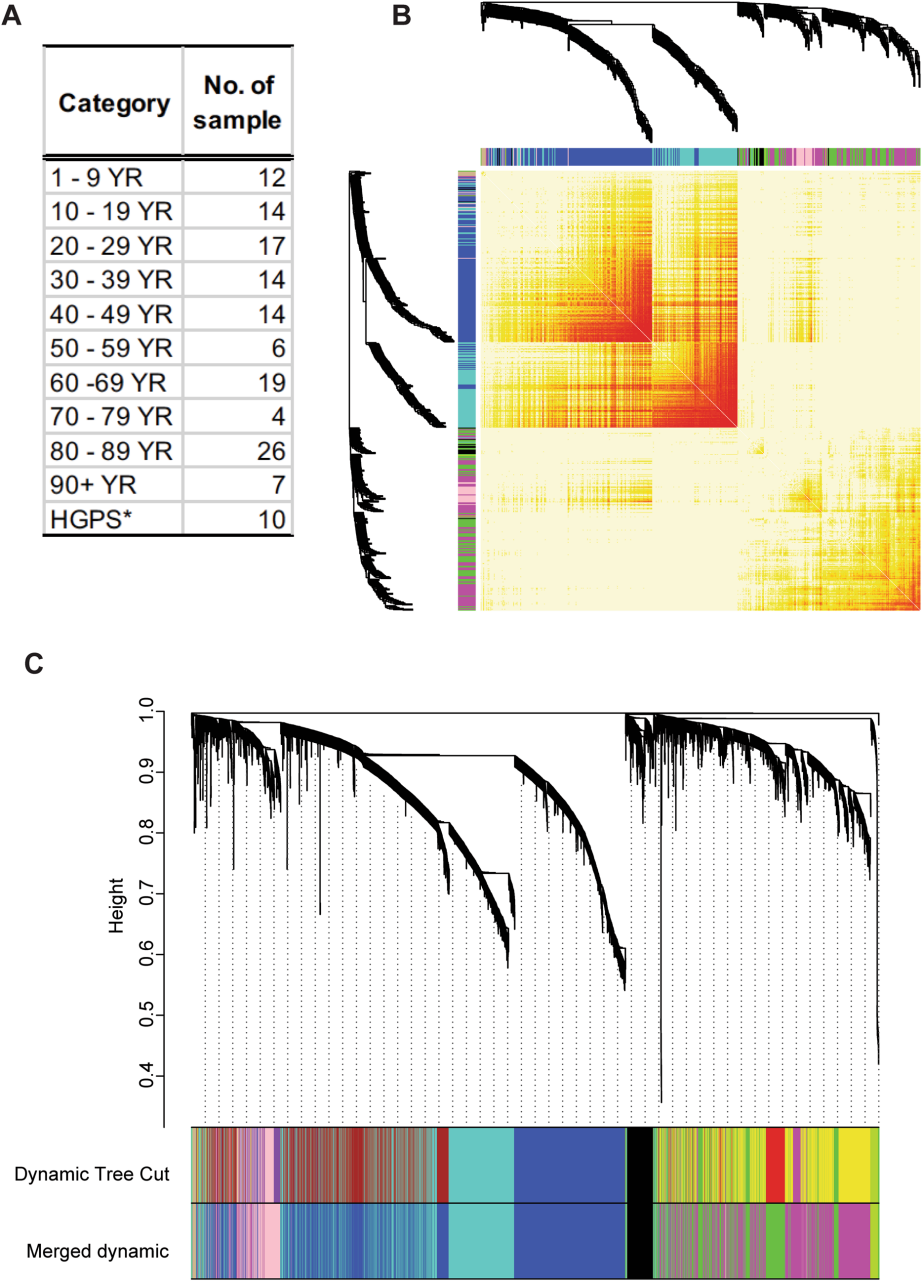
WGCNA analysis of the RNA-seq data (GSE113957), generated with young to old dermal human fibroblasts, summarized into 8 clusters of co-expressed genes. (**A**) Description of the RNA-seq data (GSE113957)^5^. It was generated with 143 human dermal fibroblasts derived from 133 healthy individuals (1–96 years) and 10 HGPS patients. (**B**) A heatmap of the topological overlap matrix (TOM) with randomly selected 500 genes, showing the pairwise relationships among genes. Each row/column corresponds to a gene. Light yellow indicates low topological overlap and darker red denotes high topological overlap, meaning high correlation in expression of the gene pair across the 143 samples. The gene dendrogram, and module assignment shown as different colour blocks are depicted along the left and top. (**C**) Identification of modules, clusters of co-expressed genes, via Dynamic Tree Cut algorithm, and subsequently merged if two modules are highly similar, resulting in 8 modules shown as different colour blocks at the bottom: Black, Blue, Green, Greenyellow, Magenta, Pink, Tan, and Turquoise.

To identify aging-dependent transcriptional changes and aging biomarkers, first, we searched the literature and found a comprehensive RNA-seq dataset (GSE113957) generated with various age groups^5^. The 143 samples for the transcriptome analysis were collected from 1 to 96-year-old healthy individuals (n = 133) and HGPS patients (n = 10) (Fig. 1A). We downloaded the processed datasets from NCBI GEO (accession: GSE113957) and filtered out the genes having low variation across the samples (“Materials and methods”) for further analysis.

WGCNA has been widely and successfully used as a data mining approach in analyzing genomics data such as expression and methylation profiles^11,12^. GSE113957 includes transcriptomes of whole age groups but does not have any replicates per sample. Thus, the conventional RNA-seq data analysis approach such as differential expression analysis is not appropriate. Rather, a large sample size and high coverage of a trait of interest (i.e., age) make it perfect for WGCNA. Therefore, we implemented WGCNA with this dataset to construct a gene co-expression network (Fig. 1B) in which pairwise correlations on expression across the 143 samples were measured. Next, we identified modules, clusters of highly correlated genes, in the gene co-expression network by applying the Dynamic Tree Cut algorithm^9^ (top colour block in Fig. 1C). Subsequently, we merged the modules if two modules were highly similar, resulting in 8 distinct modules (bottom colour block in Fig. 1C). The 8 modules were depicted with different colours in Fig. 1C, and the numbers of genes assigned to each module are as follows: Black (350), Blue (3,209), Green (1,230), Greenyellow (108), Magenta (1,824), Pink (527), Tan (43), and Turquoise (2,027).

### Identification of aging-associated modules

**Figure 2:**
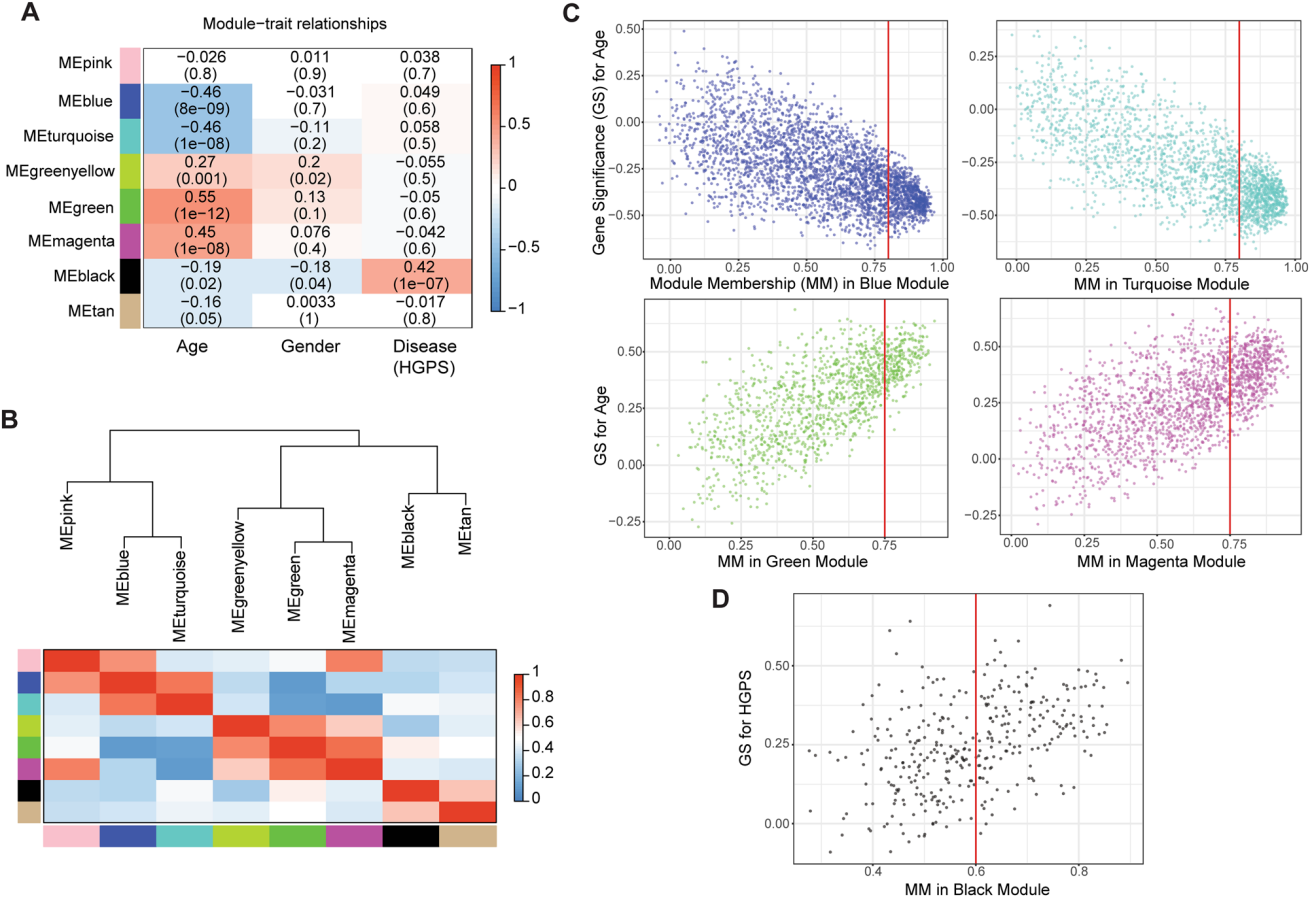
Age-dependent and HGPS-associated modules were identified by relating modules to available trait information. (**A**) A heatmap chart showing module-trait relationships. Each row is a module eigengene (ME), labelled by module colour along the left, and each column corresponds to a trait (age, gender, or disease (HGPS)). Each cell includes the corresponding correlation and p-value by correlating the module eigengenes with the matched trait outcomes. Blue indicates a negative correlation (~ − 1) and red denotes a positive correlation (~ + 1) between the module and the trait. (**B**) Hierarchical clustering of module eigengenes (MEs) to show relationships among modules. Red indicates high adjacency (~ + 1) and blue denotes low adjacency (~ 0). (**C**) Scatterplots of gene significance (GS) for age versus module membership (MM) in the Blue, Turquoise, Green, and Magenta modules. Each dot indicates one module membership (i.e., gene) assigned to the respective modules. The red vertical line in each module corresponds to a MM threshold applied to define core module memberships. (**D**) Scatterplots of GS for HGPS versus MM in the Black module.

We then asked which modules are relevant to aging by relating the modules to the available sample traits: age, gender, and disease (whether or not the donor is HGPS patient) using eigengene network methodology^9^. We found that the Blue and Turquoise modules showed significantly negative correlations with age, whereas the Green and Magenta modules revealed significantly positive correlations with age (Fig. 2A).

HGPS is a genetic disease characterized by the accelerated appearance of aging beginning in childhood^13^ and one of the most extensively studied systems to understand the significance of nuclear mechanics. This is because HGPS is caused by mutations in the *LMNA*, encoding lamin-A/C, essential structural components of the nuclear envelope^14^. *LMNA* mutations lead to the production of progerin, an abnormal version of the lamin-A protein, making the nucleus more susceptible to various DNA-damaging insults^15–17^. Progerin is also found in the cells from elderly donors, suggesting progerin-induced cellular senescence is an important factor contributing to natural aging^18^. We found one cluster of genes, the Black module, showed a significantly positive correlation with HGPS (Fig. 2A).

Clustering analysis to understand the relationship between modules revealed that the Blue and Turquoise modules were grouped and the Green and Magenta modules were clustered together (Fig. 2B), leaving these two branches apart, indicating the existence of an opposite trend in transcription change (i.e., up- or down-regulated) during human fibroblasts aging. The Black module, showing a significant link with HGPS, seemed separate from the age-dependent modules (Fig. 2B), suggesting that a distinct gene set is involved in accelerated aging caused by mutations in *LMNA*. Overall, a surprisingly large number of genes contribute to the coordinated changes in expression during human cell aging (~ 90% genes that we applied for WGCNA; ~ 30% genes in the human genome).

Next, we queried which genes are included in the aging-associated modules. To investigate the module memberships, we generated scatterplots of gene significance (GS) for age, or HGPS, versus module membership (MM) in each module (Fig. 2C,D). The GS and MM formulas are described in the original WGCNA paper^9^. Essentially, a gene having MM of 0 on the x-axis is unlikely to be part of the module, whereas a gene's MM is close to 1 indicating the gene is highly connected to the other genes within the same module. A gene with GS of 0 on the y-axis indicates that the gene is not relevant to the trait of interest, while a gene is close to -1 or 1 of GS, meaning that the gene is highly associated with the trait (i.e., age or HGPS). The negative or positive sign of GS means the expression level of the gene decreases or increases with age, respectively.

We found that the genes with a higher MM in the Blue or Turquoise module tended to have a greater negative GS for age (Fig. 2C), indicating that these genes were more relevant to increasing age and their expression decreased with age. Likewise, the genes having a greater MM in the Magenta or Green module were more relevant to aging and their expression increased with age (Fig. 2C). Also, the genes having a higher MM in the Black module appeared to be more important for HGPS and showed increased expressions with HGPS (Fig. 2D). Hence, these visual representations allowed us to pick a threshold of MM to prioritize the genes for further investigation (red vertical lines in Fig. 2C,D). This resulted in the numbers of genes down to 766 (Blue), 778 (Turquoise), 536 (Magenta), 233 (Green), and 163 (Black) and we defined these genes as "core module memberships".

To confirm if the prioritized genes (i.e., core module memberships) in the age-dependent modules would show the expression changes expected from our WGCNA, we checked the expression level of the genes by performing hierarchical clustering (Supplementary Fig. S1). The output heat map indeed exhibited two separate branches in which one cluster showed higher expressions (coloured in yellow) while the other cluster revealed lower expressions (coloured in blue) of the genes in the aged samples. Interestingly, the expression changes of the core module memberships with age were rather abruptly than gradually at the age of 86. This analysis ensured that the modules and core module memberships that we identified as age-dependent represented well the transcriptional signatures during human fibroblast aging.

### Characterization of aging-associated modules using STRING functional association network

**Figure 3:**
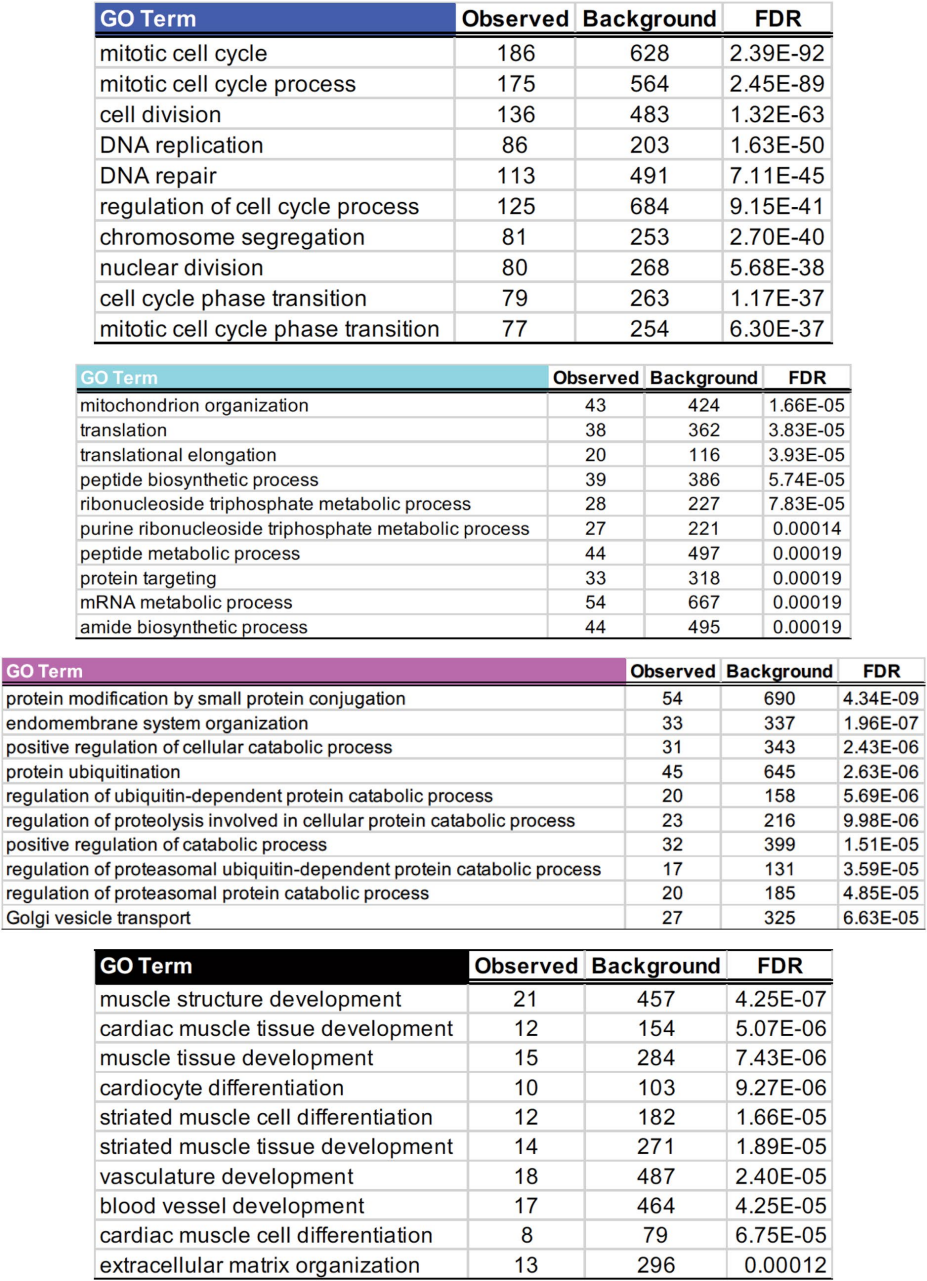
GO enrichment analysis of the STRING networks built with the core module memberships showed distinct functions in the aging-associated modules—the Blue, Turquoise, Magenta, and Black modules.

Next, we questioned the biological functions of the aging-associated modules. To characterize the modules' functions, we constructed STRING functional association networks^10^ with the core module memberships. The STRING database imports data from experimentally validated and computationally predicted protein–protein interactions, thus providing function-oriented information among the input gene list^10^. Hence, we entered the Blue, Turquoise, Magenta, Green, and Black core module memberships individually, and examined significantly enriched GO terms in the STRING networks (Fig. 3).

The highly enriched functions in the Blue STRING network were mitotic cell cycle, DNA replication, and DNA repair (Fig. 3). Because the Blue module showed a significantly negative correlation with age (Fig. 2A,C), this suggested that the mitotic cell cycle, DNA replication, and DNA repair functions declined with age. A gene with the greatest number of edges in this STRING network, defined as a functional hub gene, was *CDK1* (cyclin-dependent kinase 1), which is a key player in cell cycle regulation^20^ (Supplementary Table S2).

A significant number of genes in the Turquoise module were components of RNA polymerase, ribosome, or mitochondria (Fig. 3), suggesting these essential cellular machineries for transcription, translation, and energy generation decreased with age and the most central gene in the Turquoise STRING network was *POLR2F* (RNA polymerase II subunit F) (Supplementary Table S2).

The Magenta module having a positive correlation with age showed protein ubiquitination as the most prominent feature, and *UBE2D1* (ubiquitin-conjugating enzyme E2 D1) presented the highest number of interconnections with the rest of the Magenta module core memberships (Fig. 3, Supplementary Table S2). This presumably was attributed to the increased necessity to process misfolded proteins due to loss of proteostasis upon aging, or this may represent abnormal regulation of the ubiquitin-mediated protein trafficking in aged human cells because endomembrane system organization and Golgi vesicle transport GO terms were significant hits as well (Fig. 3).

Additionally, the Black module, significantly associated with HGPS (Fig. 2A), was characterized by muscle structure development and differentiation functions—specifically, cardiovascular functions (Fig. 3). These observations were consistent with one of the main manifestations of HGPS patients, cardiovascular alterations^19^. The functional hub gene in the Black STRING network was *MYL9* (myosin light chain 9), a key component of myosin II motor protein responsible for cell contractility in both muscle and non-muscle cells^20^.

We did not find any GO terms that were significantly enriched for the Green module. The complete GO terms significantly enriched and the hub genes in the respective STRING networks were listed in Supplementary Table S1 and S2. Taken together, our GO enrichment analysis with the STRING functional networks provided a systematic overview of the genome-wide transcriptional changes upon aging.

### Experimental validation of selected genes in aging-associated modules using RT-qPCR

**Figure 4:**
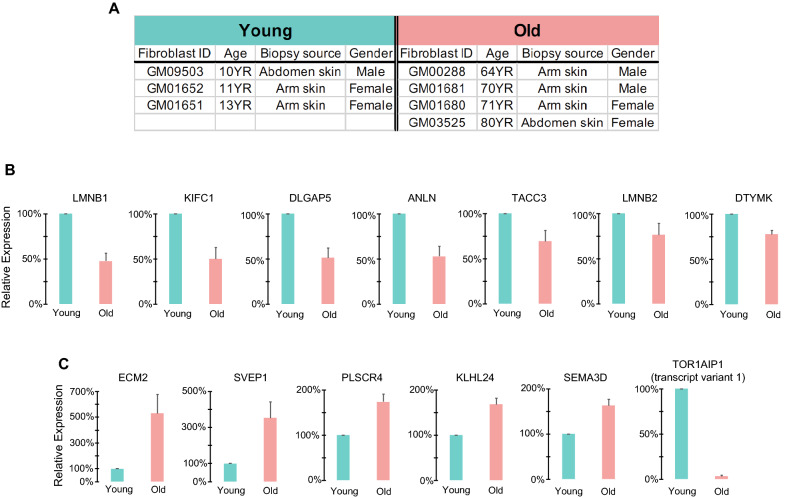
The selected genes from the Blue and the Green modules revealed notable differences in expression between young and old fibroblasts. (**A**) Details on the primary human dermal fibroblasts used in the RT-qPCR validation. (**B**,**C**) RT-qPCR validation of genes selected from the Blue (**B**) and Green modules (**C**). The error bars indicate the standard error of the mean, calculated with at least three and at most seven replicates.

In addition to our bioinformatics analysis, we set up an experimental approach with primary skin fibroblasts derived from young and old human donors (aged 10–13 years and aged 64–80 years, respectively) (Fig. 4A) to validate some of the core module memberships. To select the candidates for validation, we took account of (1) the degree of expression change between young and old groups by averaging the expression values of 1–29 years and 60–96 years, respectively, in the RNA-seq data, and (2) GS score (i.e., GS × − log_10_(*p-value*)). Interestingly, a considerable number of genes that play a role in defining the mechanics of cells, including *ANLN*, *LMNB1*, *LMNB2*, *TOR1AIP1*, *ITGB8*, *ECM2*, and several more, were selected in our list (Supplementary Table S3). We cultured the young and aged fibroblasts, isolated RNA, and synthesized cDNA to measure transcription levels of the selected genes via qPCR (Supplementary Table S3).

*LMNB1*, encoding lamin-B1, an established cellular aging marker^21^, showed a 50% reduction in expression for the aged fibroblasts (Fig. 4B). Several genes in the Blue module revealed a significant reduction in expression in aged fibroblasts compared to young fibroblasts (p-value < 0.1), which include *KIFC1* (kinesin family member C1), *DLGAP5* (disks large-associated protein 5), *ANLN* (anillin), *TACC3* (transforming acidic coiled-coil-containing protein 3), *LMNB2* (lamin-B2), and *DTYMK* (deoxythymidylate kinase) (Fig. 4B).

Moreover, several genes selected from the Green module, such as *ECM2* (extracellular matrix protein 2), *SVEP1* (sushi, von Willebrand factor type A, EGF and pentraxin domain-containing protein 1), *PLSCR4* (phospholipid scramblase 4), *KLHL24* (kelch-like family member 24), and *SEMA3D* (semaphorin-3D) revealed a significant increase in expression for the fibroblasts from elderly donors (Fig. 4C).

Surprisingly, *TOR1AIP1* (torsin-1A-interacting protein1, also known as *LAP1B*, lamina-associated polypeptide 1), selected from the Green module, showed the robustness and specificity in differentiating young and old fibroblasts (Fig. 4C). *TOR1AIP1* expressed highly in the young fibroblasts whereas rarely expressed in the aged fibroblasts regardless of inter-individual variabilities in expression and multiple biological replicates (Fig. 4C), which involves the variations in the cell passage number, the cell density in the culture vessels, and the proliferating capacity (i.e., the ratio of the senescent cells to the proliferating cells). These variations were not trivial in the RT-qPCR readout for the other genes, resulting in lesser magnitudes of the expression change between young and old samples, and higher error bars in the graphs, compared to the ones shown in the *TOR1AIP1* graph (Fig. 4B,C).

In addition to the extreme specificity of TOR1AIP1 in distinguishing chronological age, one puzzling question was that the direction of the expression change was opposite because *TOR1AIP1* was selected from the Green module which was predicted having a positive correlation with age. We solved this by confirming the existence of two transcript variants of *TOR1AIP1*—NM_001267578.2 (variant 1) and NM_015602.4 (variant 2)^22,23^, and the fact that our RT-qPCR primers annealed and amplified specifically for the transcript variant 1, NM_001267578.2 (Supplementary Fig. S4B). Yet, the RNA-seq data that we utilized for WGCNA reported a consolidated gene-level expression (Supplementary Fig. S4A), not the expression of individual transcripts^5^.

*TOR1AIP1* encodes an integral membrane protein located at the inner nuclear membrane, interacting with lamin-A/C and lamin-B, and it is essential for nuclear membrane integrity^14^. Besides of its significant role in nuclear mechanics, age-dependent expression of different transcript variants that utilize an alternate splice site (Supplementary Fig. S4B) seems an interesting hypothesis to be tested. Hence, we investigated further if there is a biased expression of *TOR1AIP1* transcript variant 1 depending on donor’s age, and our analysis was described in the Discussion section. The complete set of genes that we tested by RT-qPCR, along with the statistical test on RT-qPCR readout, were listed in Supplementary Table S3.

## Discussion

Here, we carried out WGCNA with transcriptome data generated with human dermal fibroblasts derived from comprehensive age groups^5^. We uncovered the presence of aging-associated modules, clusters of co-expressed genes. We further characterized the aging-associated modules, focusing on their functions by constructing STRING functional association networks with the prioritized genes in the modules. We found that the mitotic cell cycle, DNA repair and replication, mitochondrion organization, and translation were negatively correlated; whereas protein ubiquitination and vesicle-mediated intracellular protein transport were positively correlated with age. Additionally, we presented functional hub genes—*CDK1* (Blue), *POLR2F* (Turquoise), *SNAP23* (Green), *UBE2D1* (Magenta), and *MYL9* (Black) in the respective STRING networks. These hub genes had the largest number of interconnection with the rest of the genes within the respective network, and as such, these genes are likely to have the greatest impact on the functional network. Therefore, the hub genes could serve as a potential target for reverse genetics tools to induce module-wise functional deterioration in the cells, which will help to dissect the multifaceted aging process. Lastly, our attempt to validating our WGCNA analysis experimentally enabled us to present novel candidates for aging biomarkers, namely *KIFC1*, *DLGAP5*, *ANLN*, *ECM2*, *SVEP1*, and *TOR1AIP1*.

Our study generated several intriguing perspectives in mechanobiology of aging, described as follows.

Cellular senescence, losing the proliferation capacity of cells, is characterized by the cessation of cell division, and it is one of the antagonistic hallmarks of aging—defensive responses to various cellular stresses^1,24,25^. Here, we found that the mitotic cell cycle-related genes coordinately down-regulated with age. Misregulation in the mitotic gene expression during aging has been reported in several studies^26,27^. One recent study^27^ found that the repression of FoxM1 with aging, which is a key transcription factor regulating the expression of cell cycle genes, was a direct cause of the downregulation of mitotic genes and the transcription signature of cellular senescence, thereby dictating cellular phenotypes associated with aging. A substantial proportion of the mitosis gene set, which was found to be significantly downregulated in the cells from an elderly donor (i.e., 87 years) in that study^27^, and our Blue module memberships were overlapping, ensuring our WGCNA analysis and findings were reliable.

Moreover, our experimental validation via RT-qPCR indicated that cytokinesis, where coordinated mechanical forces are required to ensure proper sister chromatid segregation^28^, maybe a stage more susceptible to age-dependent mitotic decline. This was supported by our observation that more prominent expression change was revealed for the cytokinesis genes, encoding kinetochore-interacting proteins (*KIFC1*, *DLGAP5*, and *TACC3*) and an actin-binding protein enriched at contractility ring (*ANLN*) during cytokinesis (Fig. 4B). The subsequent GO analysis with the further prioritized genes by applying a threshold of greater than twofold decrease in expression for the cells from elderly donors (60–96 years) compared to that from the young donors (1–29 years) showed significant enrichment of cytokinesis-related terms (Supplementary Fig. S3). This suggested that cytokinesis gene downregulation was one of the appreciable transcriptional signatures with aging.

Mutations in *TOR1AIP1* result in muscular dystrophy or progressive dystonia with cerebellar atrophy^29^ and the cell-level phenotypes due to the loss-of-function mutations are abnormal nuclear morphologies such as low lamin-A/C level at the nuclear envelope and abnormal cytoplasmic channels spanning the nucleus^29^. Because the loss of *TOR1AIP1* leads to the premature aging phenotype, and we found a remarkable specificity in differentiating cells' chronological age (Fig. 4C), it was one of our great interests. Hence, we attempted to re-align raw RNA-seq data (BioProject Accession: PRJNA454681)^30^ to the human reference transcriptome (GRCh38_latest)^31^ using Kallisto^32^. To test our hypothesis that *TOR1AIP1* transcript variant 1 is rarely expressed for the aged fibroblasts, we quantified *TOR1AIP1* transcript variant 1 and 2 separately depending on the donor’s age (Supplementary Fig. S4C). The mean expression level of *TOR1AIP1* transcript variant 1 did not seem to decrease with age (left, Supplementary Fig. S4C). Yet, we found a group of samples localized at y-axis = 0 across all age groups including HGPS, indicating the existence of the cells not expressing *TOR1AIP1* transcript variant 1 at all (Supplementary Fig. S4C). Considering the population probability of no expression of *TOR1AIP1* transcript variant 1 was 0.38 (38%, 54 out of 143 fibroblast lines), this probability appeared not equal across the age groups. It was noteworthy that the probability was higher for the 80–96 years and HGPS groups (45% and 60% respectively, Supplementary Fig. S4D). This was probably why we observed almost no expression of the *TOR1AIP1* transcript variant 1 for the aged fibroblasts (n = 4) in our RT-qPCR validation (Fig. 4C), even though we chose the fibroblast lines independently from the RNA-seq data. It will be interesting to see if the biased usage of transcript variants with aging is widespread across the human genome.

ECM’s composition, arrangement, thickness, stiffness, and rates of remodeling and degradation are precisely regulated to provide proper biochemical and biophysical supports to the surrounding cells^2,33^. Aging-associated alteration in ECM forms the tissue microenvironment prone to the transformation of a cell, such as cancerous or senescent. The senescent cells expressing a variety of secreted proteins, such as inflammatory cytokines and chemokines, growth factors, and proteases (i.e., senescence-associated secretory phenotype, SASP) disturb the balanced biochemical signalling, developing the aged microenvironment, which further contributes to aging^34,35^. Here, we found the expression of two genes encoding ECM proteins, *ECM2* and *SVEP1*, was surprisingly high for the old fibroblasts (Fig. 4C). The expression of *ECM2* and *SVEP1* was the highest for the GM01681 fibroblasts (70-year-old donor), and this cell line showed the least proliferating capacity, measured by the percentage of Ki-67 positive cells, a cell proliferation marker (Supplementary Fig. S2A,B). This suggests that *ECM2* and *SVEP1* may participate in cellular senescence presumably by affecting the aged microenvironment. It will be intriguing to test if the reduction of *ECM2* or *SVEP1* in aged cells possibly restore cells’ proliferating capacity.

HGPS is a premature aging disorder characterized by accelerated cardiovascular disease with extensive fibrosis^36^. The progressive deposition of collagen fibers and an increased number of and thicker actin filaments are the manifestations of tissue fibrosis, and aging is a high-risk factor for the tissue fibrosis, namely in lung and heart^2^. Here, we found that the Black module was significantly associated with HGPS (Fig. 2A). The Black module included multiple genes that function in the actomyosin contractility (*MYL9*, *MYH11*, *ACTA2*, *ACTG2*, *CALD1*, *LMOD1*, *TAGLN*, and *MYOCD*) and ECM organization (*COL4A1*, *COL4A2*, *COL4A5*, *COL11A1*, and *ITGA7*) (Supplementary Table S2). The expression of these genes was abnormally high for the HGPS-derived fibroblasts compared to that for the any other age groups (i.e., early (1–29 years), middle (30–59 years), and late (60–96 years) age) (Supplementary Fig. S5), suggesting these cells, having defects in nuclear mechanics due to mutations of the *LMNA* gene, tend to show excessive actomyosin and collagen gene signatures, possibly leading to an increase in local tissue stiffness and fibrosis.

All in all, our study presents a transcriptome-based quantitative framework for assessing human dermal fibroblast aging as well as interesting transcription signatures involved in ECM, cell, and nuclear mechanics, which will be useful for aging and rejuvenation studies.

## Materials and methods

### Weighted gene co-expression network analysis (WGCNA)

GSE113957^5^ was downloaded from NCBI GEO and filtered out the genes with low variation by applying a threshold of 1 of standard deviation across the samples. This threshold was stringent enough to filter out low expressed genes, which resulted in 9318 genes that were subsequently used for WGCNA. The detailed parameters that we applied for WGCNA as follows:

- network type = signed
- soft power = 13
- module identification method = dynamic tree cut
- minimum module size = 30
- the threshold to merge modules with a high similarity = 0.2

Default values were used unless specified. Gene significance (GS) for age or HGPS, and Module Membership (MM) were calculated according to the original article describing WGCNA^9^.

### STRING functional association network analysis

The core module memberships were entered into the STRING Search tool^10^ and a high confidence interaction score (0.7) was applied to the output networks to maintain reliable functional interactions. The significantly enriched Gene Ontology (GO) Biological Process terms in the STRING network were exported and trimmed by removing too general terms, which have greater than 700 genes assigned (Background > 700) to reduce redundancy of similar GO terms. To determine functional hub genes in the STRING network, we counted the number of edges of the individual nodes (i.e., genes) in the respective network, and sorted the numbers in descending order.

### Primary human skin fibroblasts

Seven primary human skin fibroblast lines—GM09503 (passage frozen (P), P3), GM01652 (P11), GM01651 (P14), GM00288 (P9), GM01681 (P12), GM01680 (P12), and GM03525 (P7) —were purchased from the Coriell Institute (Camden, NJ, USA). The fibroblasts were cultured in MEM (Gibco) supplemented with 15% (v/v) heat inactivated FBS (Gibco), 1% (v/v) MEM non-essential amino acids (Gibco), and 1% (v/v) Pen-Strep (Gibco) in the incubator (37 °C, 5% CO_2_). The cells within 7 passages from the initial P were used for RT-qPCR assays.

### RNA isolation and RT-qPCR

RNA was prepared using RNeasy Plus Mini Kit (QIAGEN) and cDNA was synthesized using SuperScript IV reverse transcriptase (Invitrogen) according to the manufacturer’s protocols. Quantitative real-time PCR was carried out using SsoAdvance Universal SYBR Green Supermix (Bio-Rad) and Bio-Rad CFX 96 Real-Time PCR Detection System according to the manufacturer’s protocols. The relative expression of a gene was calculated using the delta-delta Ct (ddCt) method. Firstly, the delta Ct (dCt) of individual genes was obtained by subtracting the Ct of a housekeeping control gene (*HPRT1*) from a target gene’ Ct. Then, the dCt values for young (n = 3) and for old (n = 4) fibroblasts were averaged, and subsequently, the mean dCt_young_ and mean dCt_old_ were normalized such that the mean dCt_young_ was equal to 0—i.e., ddCt_young_ = 0. The relative expression was 2^(−ddCt)^, formatted as percentage. Paired t-test was performed on the mean dCt_young_ and mean dCt_old_ values that were obtained from three to seven replicate experiments to see if the gene expression is different between young and old fibroblasts. The p-values from the statistical testing and the primer sequences are listed in Supplementary Table S3. The primers were designed using NCBI Primer-BLAST.

## Supplementary information

Supplementary Information 1.

Supplementary Information 2.

Supplementary Information 3.

Supplementary Information 4.

Supplementary Information 5.

Supplementary Information 6.
