# Supplementary material for: Analysis of transcriptional modules during human fibroblast ageing
Source: Sci Rep. 2020 Nov 5;10:19086. doi: 10.1038/s41598-020-76117-y (PMC7645754; doi:10.1038/s41598-020-76117-y)

Supplementary Materials

Analysis of transcriptional modules during human fibroblast ageing

Yaelim Lee and G.V. Shivashankar

Supplementary Figure S1

Supplementary Figure S2

Supplementary Figure S3

Supplementary Figure S4

Supplementary Figure S5

Supplementary Figure S1

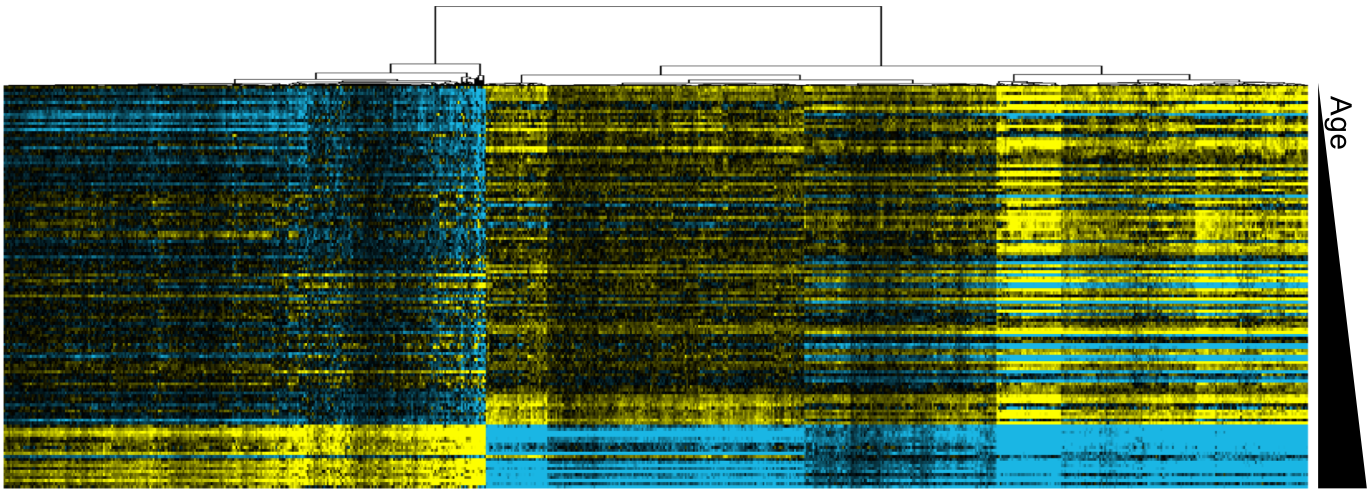

Supplementary Figure S2

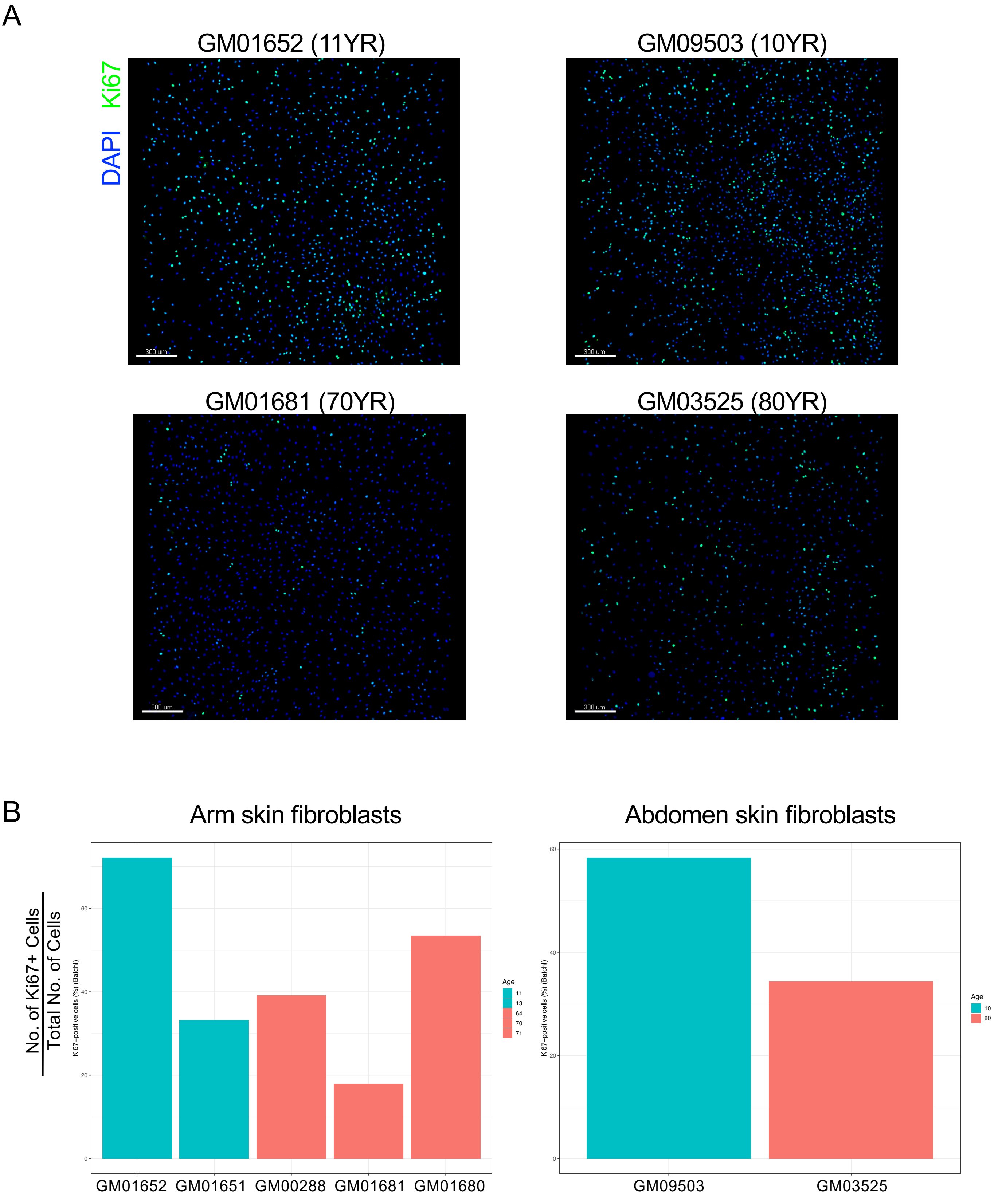

Supplementary Figure S3

GO Term	Adjusted P-value	Odds Ratio	Combined Score
sister chromatid segregation	2.17E-13	98.68	3583.09
mitotic sister chromatid segregation	4.02E-18	59.91	2911.00
mitotic nuclear division	4.08E-15	56.90	2331.55
mitotic spindle organization	2.24E-13	52.16	1907.13
mitotic metaphase plate congression	3.57E-10	65.28	1872.27
establishment of chromosome localization	1.18E-05	107.96	1834.91
microtubule cytoskeleton organization involved in mitosis	3.11E-10	63.80	1817.06
metaphase plate congression	3.63E-10	63.80	1817.06
regulation of chromosome segregation	9.41E-08	72.60	1627.11
regulation of mitotic sister chromatid separation	2.12E-05	93.57	1530.15
regulation of attachment of spindle microtubules to kinetochore	4.91E-04	105.26	1353.83
attachment of mitotic spindle microtubules to kinetochore	5.87E-04	95.69	1200.47
kinetochore organization	6.91E-04	87.72	1075.37

Supplementary Figure S4

A

Age group	No. of samples for averaging
1 - 9 YR	12
10 - 19 YR	14
20 - 29 YR	17
30 - 39 YR	14
40 - 49 YR	14
50 - 59 YR	6
60 -69 YR	19
70 - 79 YR	4
80 - 89 YR	26
90+ YR	7

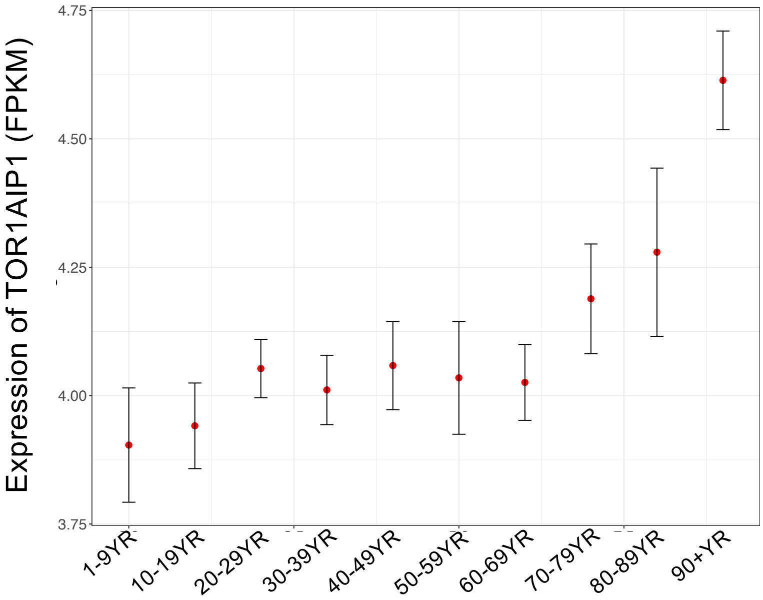

B

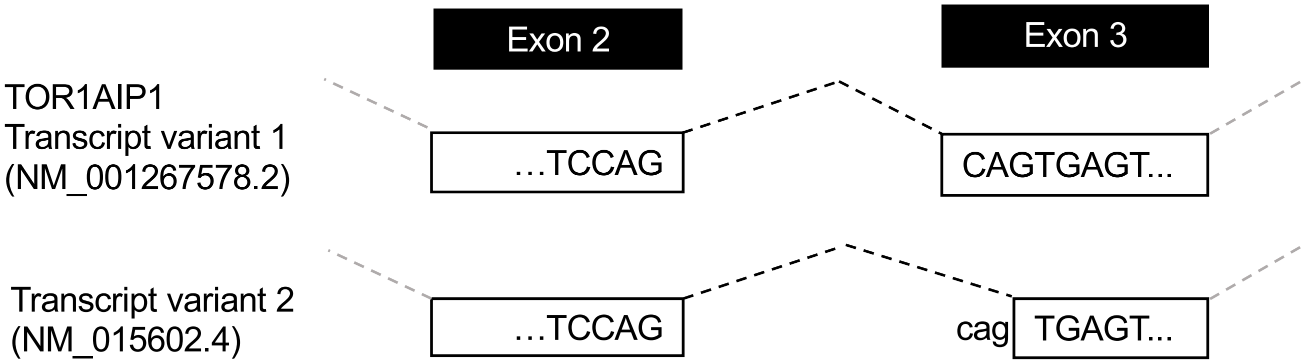

C

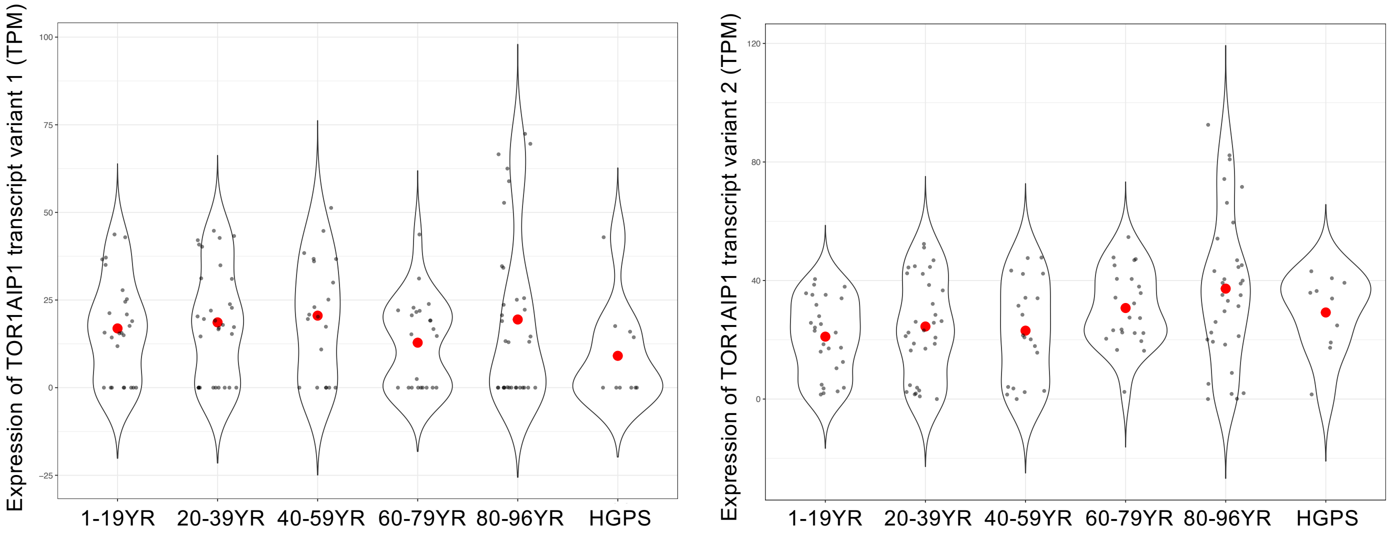

D

Age Group	1-19YR	20-39YR	40-59YR	60-79YR	80-96YR	HGPS	Total
No. of samples NOT expressing TOR1AIP1 transcript variant 1	8	10	6	9	15	6	54
No. of samples	26	31	20	23	33	10	143
	31%	32%	30%	39%	45%	60%	38%

Supplementary Figure S5

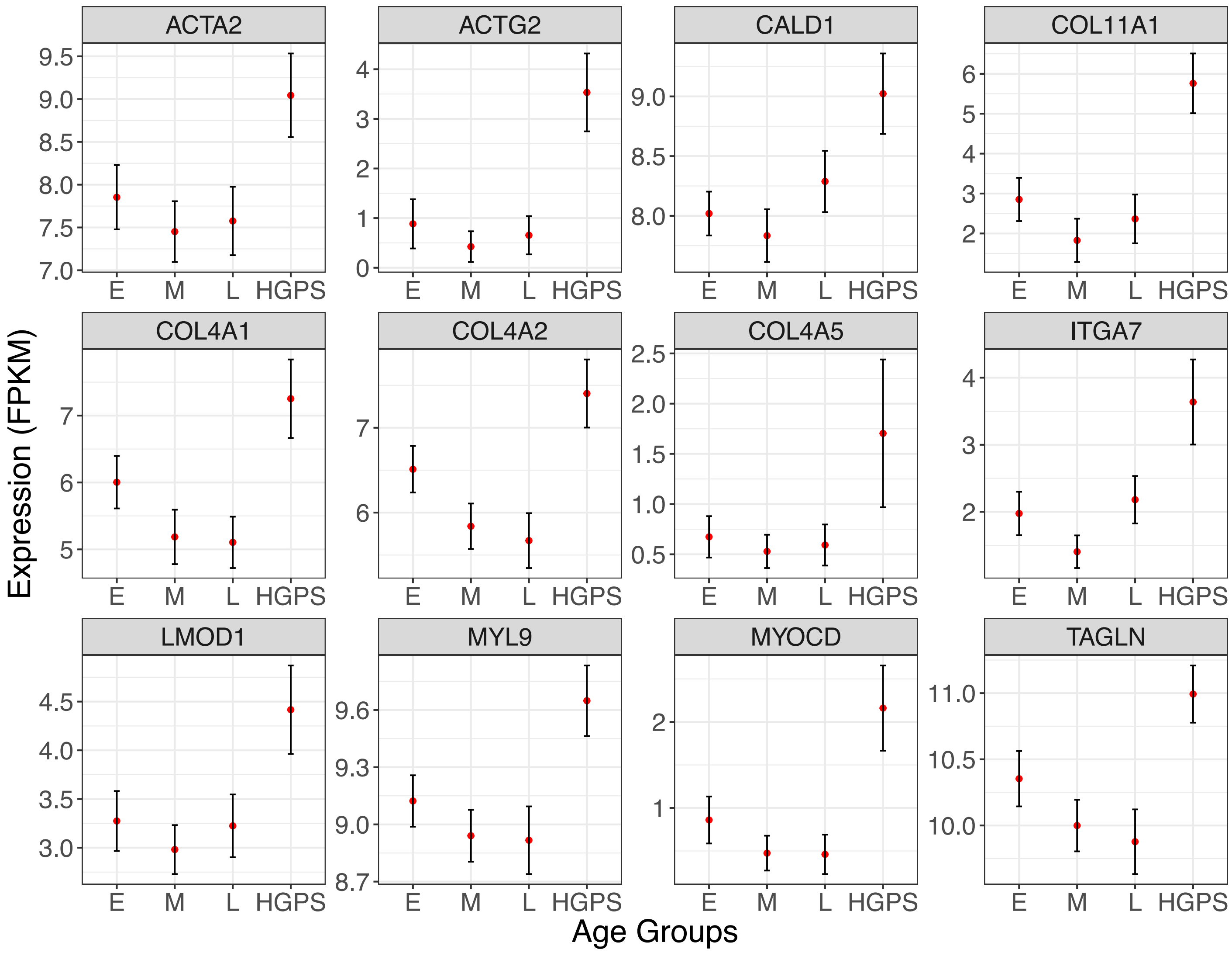

Supplement: Supplementary file 4 — Supplementary Information 4. [file 41598_2020_76117_MOESM4_ESM.pdf]
