# Supplementary material for: Analysis of transcriptional modules during human fibroblast ageing
Source: Sci Rep. 2020 Nov 5;10:19086. doi: 10.1038/s41598-020-76117-y (PMC7645754; doi:10.1038/s41598-020-76117-y)
Supplement: Supplementary file 5 — Supplementary Information 5. [file 41598_2020_76117_MOESM5_ESM.pdf]

Supplementary Materials and Methods

Immunofluorescent (IF) staining & Confocal imaging

Cells were fixed using 4% paraformaldehyde (PFA) in PBS for 15 minutes at room temperature (RT) and subsequently rinsed with PBS twice. Cells were permeabilized for 15 minutes with 0.3% Triton X-100 in PBS. Blocking was done with 5% BSA and 0.3% Triton X-100 in PBS for at least 2 hours at RT. Cells were incubated with the Ki67 antibody (Cell Signaling Technology, Cat.# 9449) diluted in AB dilution buffer (2% BSA and 0.2% Triton X-100 in PBS) overnight at 4°C. Next, cells were washed with three different IF washing buffers for 15 minutes each: (i) 0.2% Triton X-100 in PBS, (ii) 0.2% Tween 20 in PBS, (iii) PBS. Then, cells were incubated with Alexa flour 488-conjugated secondary antibodies and Phalloidin diluted in the AB dilution buffer for 1 hour at RT, followed by washing with the three IF washing buffers for 15 minutes each. The nuclei were stained with NucBlue Live Ready Probes (Molecular Probes) in PBS for 10 minutes at RT before imaging. The stained samples were scanned using the Nikon A1Rsi confocal microscope (Nikon) with a 20x objective. To capture many numbers of cells for quantification (*i.e.,* No. of cells > 1000), large images were taken by stitching multiple fields of view (2.25 mm x 2.25 mm). The microscopy images were analyzed by custom-written codes in MATLAB. The Ki67-positive cells were defined by applying a threshold of median Ki67 intensity value of all nuclear Ki67 intensities per imaging session, which were captured with the same image acquisition parameters.
