# Supplementary material for: Analysis of transcriptional modules during human fibroblast ageing
Source: Sci Rep. 2020 Nov 5;10:19086. doi: 10.1038/s41598-020-76117-y (PMC7645754; doi:10.1038/s41598-020-76117-y)
Supplement: Supplementary file 6 — Supplementary Information 6. [file 41598_2020_76117_MOESM6_ESM.docx]

**SUPPLEMENTARY FIGURES**

**Supplementary Figure S1.** Hierarchical clustering of expressions of the top 10% Blue, Turquoise, Green, and Magenta module memberships. Each row corresponds to a RNA-seq sample (GSE113957), sorted by donors' age, and each column corresponds to a gene. The top 10% module memberships were selected based on MM. Blue indicates low expression and yellow depicts high expression.

**Supplementary Figure S2.** Ki-67 Immunofluorescence (IF) staining of young and old fibroblasts. (A) Representative microscopy images of Ki-67 IF staining of young (GM01652 and GM09503) and old (GM01681 and GM03525) fibroblasts. DAPI nuclear counter staining and scale bar = 300μm. (B) Quantification of the Ki-67 IF staining.

**Supplementary Figure S3.** GO analysis of selected genes from the Blue module upon applying a threshold of > 2-fold decrease in expression between early and late age groups (0YR – 29YR and 60YR – 96YR, respectively) using Enrichr ^37^.

**Supplementary Figure S4**. Expression of TOR1AIP1 transcript variants 1 and 2 in young to old dermal fibroblasts.

(A) The gene-level expression of TOR1AIP1 in the RNA-seq data (GSE113957) depending on the donor' age. The gene-level expression of TOR1AIP1 was averaged over a 10YR interval as shown in the table (Left). A red dot and black bar depict the mean expression of TOR1AIP1 and a 95% confidence interval of the mean at the specified age group. (B) A schematic diagram showing the 3 nucleotides difference in splicing between TOR1AIP1 transcript variants 1 and 2 (NM_001267578.2 and NM_015602.4). The 3 nucleotides, CAG, are located at the 5' end of the third exon. The lowercase letters, cag, indicate the nucleotides reside in the intron. (C) Expressions of TOR1AIP1 transcript variant 1 (Left) and variant 2 (Right) depending on the donor' age (20YR interval) and HGPS groups. Black dots indicate individual RNA-seq samples and a red dot depicts the mean expression of the TOR1AIP1 transcript at the specified age/HGPS group. (D) A numeric summary table showing the proportion of samples having no expression of TOR1AIP1 transcript variant 1 across the age/HGPS groups.

**Supplementary Figure S5.** Expressions of actomyosin contractility and collagen genes, selected from the Black module. E, M, L, and HGPS on the X-axis indicate early- (1-29YR), middle- (30-59YR), late- (60-96YR) aged, and HGPS groups. The expression values reported in the RNA-seq data (GSE113957) were averaged according to the specified groups. Red dots and black bars indicate mean and 95% confident interval of the mean.

**SUPPLEMENTARY TABLES**

**Supplementary Table S1.** The complete GO terms significantly enriched in the respective STRING networks

**Supplementary Table S2.** The complete hub genes in the respective STRING networks

**Supplementary Table S3.** List of genes tested by RT-qPCR, p-values from statistical testing on the RT-qPCR readout, and sequences of qPCR primers
